# Genetic and functional analysis of a Li Fraumeni syndrome family in China

**DOI:** 10.1038/srep20221

**Published:** 2016-01-28

**Authors:** Huaying Hu, Jingping Liu, Xinbin Liao, Shuju Zhang, Haibo Li, Renbin Lu, Xianfeng Li, Wei Lin, Minji Liu, Zanxian Xia, Guoliang Qing, Jia-Da Li

**Affiliations:** 1 The State Key Laboratory of Medical Genetics and School of Life Sciences, Central South University, Changsha, Hunan 410078, China; 2 Department of Neurosurgery, Xiangya Hospital, Central South University, Changsha, Hunan 410080, China; 3 Department of Pathology, Xiangya Hospital, Central South University, Changsha, Hunan 410080, China; 4 Medical Research Institute, Wuhan University, 185 Donghu Rd, Wuhan, Hubei 430071, China; 5 The Molecular Cytogenetics Laboratories, Beijing Jiaen Hospital, Beijing, 100191, China

## Abstract

Li Fraumeni syndrome (LFS) is a rare familial cancer predisposition syndrome with autosomal-dominant inheritance, occurring as frequently as one in 5,000–20,000 individuals. However, no LFS case has been reported from mainland China although it constitutes one quarter of population on earth. In this study, we identified, to our best knowledge, the first Li Fraumeni syndrome family in China. Six family members were affected with various tumors. A *TP53* mutation (c.730G > A; p.G244S) co-segregated with the tumor phenotype within this family. Functional analysis indicated that G244S mutation disrupted the transactivity, DNA-binding and cell growth inhibition activity of p53 protein. Two available tumor samples (medulloblastoma and choroid plexus papilloma) underwent large rearrangement in the chromosomes and loss of wild-type *TP53*. Our data warranted further studies on the prevalence of germline *TP53* mutation in various tumor patients in China.

Li Fraumeni syndrome (LFS), first introduced by Li and Fraumeni in 1969, is a rare familial cancer predisposition syndrome with autosomal-dominant inheritance^1^,^2^. The clinical definition of LFS includes a proband with a sarcoma under the age of 45 years, a first-degree relative with a cancer prior to the age of 45 years, and a first- or second-degree relative with any cancer before 45 years or a sarcoma at any age^3^. The major tumors in LFS include soft tissue and bone sarcomas, breast cancer, brain tumors, adrenocortical carcinomas, and acute leukemia, *et al*.^4^,^5^,^6^,^7^.

The risk of individuals with LFS to develop any invasive cancer is ~50% by age 30 and is 90% by age 70^8^. Females with this syndrome have almost a 100% lifetime risk of developing cancer, whereas the risk for affected males is 73%^9^. Individuals with LFS are also prone to develop second, third, even fourth malignant neoplasms, with the highest risk of additional cancers being in those diagnosed with their first cancer during childhood.

Approximately 70% of families with LFS have a mutation in the *TP53* gene^10^. *TP53* encodes the p53 tumor suppressor, a critical transcription factor that promotes cell-cycle arrest, apoptosis, and DNA repair in response to cellular stresses such as exposure to ionizing radiation^11^,^12^,^13^,^14^,^15^,^16^,^17^,^18^,^19^,^20^. Mutations that interfere with the transcriptional activity of p53 reduce its growth suppressive functions. Moreover, mutant p53 proteins may also acquire oncogenic gain of function^21^,^22^. For instance, mice with one mutant allele equivalent to human R175H, R273H, or R248Q showed more spontaneous carcinomas, sarcomas and lymphomas than heterozygous or null (*TP53*^*+/−*^ or *TP53*^−/−^) mice^23^,^24^,^25^,^26^.

LFS may be as frequent as one in 5,000–20,000 individuals^27^. Until now, there are more than 500 LFS families have been identified worldwide^10^. Although it constitutes one quarter of population on earth, no LFS has been reported from mainland China. In this study, we identified a family with multiple types of tumors. A *TP53* mutation (c.730G > A; p.G244S) was identified in the patients. This mutation led to functional disruption of p53 protein.

## Results and Discussion

### Clinical findings of the family

**Figure 1:**
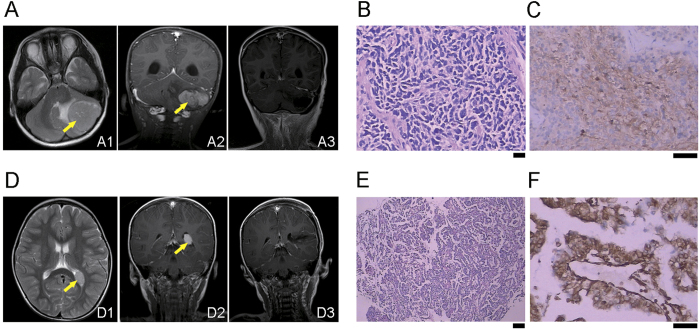
The Magnetic Resonance Imaging (MRI) and histology of tumors from the proband (III: 4) and her brother (III: 5). (**A**) The MRI of the proband’s tumor (indicated with arrows) located in the left cerebellar hemisphere (A1-A2). No recurrence was detected at 18 months post-surgery (A3). (**B**) The haematoxylin and eosin (H&E) staining of the proband’s tumor sample. (**C**) The immunohistochemistry of proband’s tumor sample stained with neuron specific enolase (NSE) antibody. (**D**) The MRI of the III: 5’s mass (indicated with arrows) in the posterior of left lateral ventricle (D1-D2). No recurrence was detected at 18 months post-surgery (D3). (**E**) The H&E staining of the III: 5′s tumor sample. (**F**) The immunohistochemistry of III: 5′s tumor sample stained with pan cytokeratin antibody. Scale bar: B and D: 200 μm; C and E, 100 μm.

In 2014, the proband (III: 4, a 5-year-old girl) was diagnosed with a tumor located in the left cerebellar hemisphere (Fig. 1A1, 2) in the Department of Neurosurgery at Xiangya Hospital, Central South University of China. The mass was removed surgically and diagnosed as classical medulloblastoma pathologically (Fig. 1B,C). The proband undertook a standard radiotherapy after surgery, and no recurrence was detected after 18 months (Fig. 1A3).

Soon later, the proband’s younger brother (III: 5, a 3-year-old boy), was accidentally diagnosed with a mass in the posterior of left lateral ventricle (Fig. 1D1, 2). After a craniotomy, it was proved to be choroid plexus papilloma (Fig. 1E,F). The mass was removed by surgery and no recurrence was detected after 18 months (Fig. 1D3).

**Figure 2:**
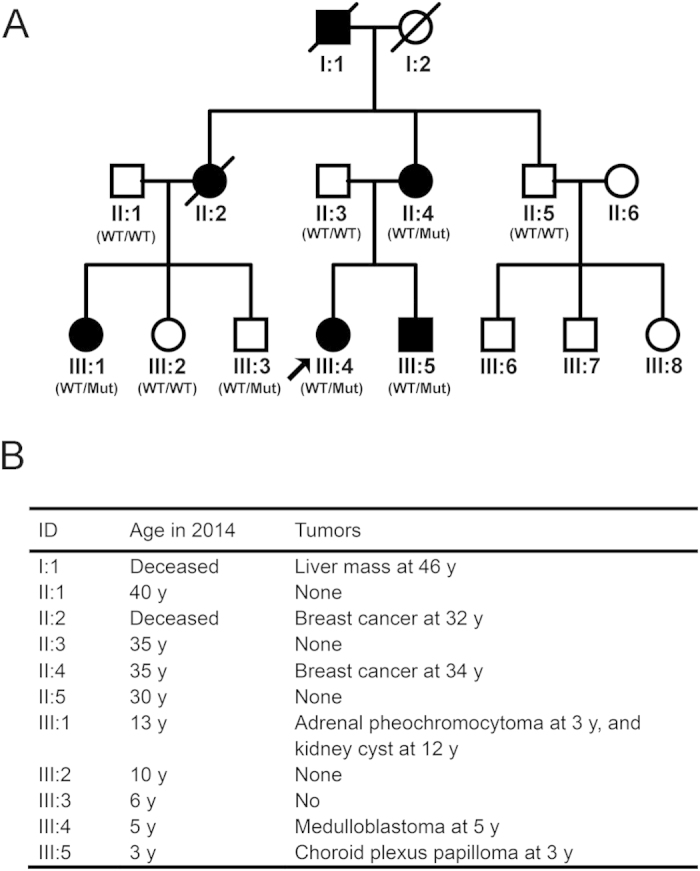
The patient information. (**A**) The pedigree of this family. Subjects in the family are identified by the Roman and Arabic numerals below the symbol, in which the Roman numerals denote the generations. Open symbols = unaffected; filled symbols = affected; symbols with a diagonal line = deceased subjects; squares = male; circles = female; arrow = the proband; WT = wild-type *TP5*3 allele; Mut = G244S mutant *TP5*3 allele. (**B**) The patient information including age, tumor type and tumor onset age.

The fact that two individuals in a family carried tumors at very young age caught our attention; we therefore surveyed the tumor history in this family. As shown in Fig. 2A,B, the proband’s mother (II: 4) was diagnosed with ductal carcinoma *in situ* by breast needle biopsy at the age of 34 y in 2013. No pathogenic mutation was found in genes *BRCA1* or *BRCA2* by Sanger sequencing. She underwent a radical operation later and was well in the two-year follow-up. The proband’s aunt (II: 2) underwent a modified radical mastectomy and was diagnosed as occult breast cancer in 2010; she died of distant metastasis in 2013 despite of standard chemotherapy. The tumor samples from II: 2 was demonstrated to be negative in ER and PR by immunohistochemistry. The oldest daughter (III: 1) of patient II: 2 underwent two surgical treatments. She was diagnosed with adrenal pheochromocytoma at 3 y, and renal cyst at 12 y. Moreover, the proband’s maternal grandfather died with a liver mass at his 40 s. Accordingly, the tumors in this family were aggregated and likely to be Li Fraumeni syndrome.

### *TP53* mutation in the family

**Figure 3:**
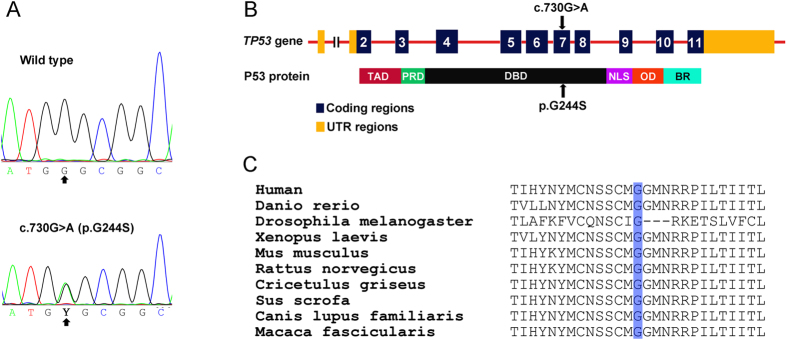
G244S mutation of the *TP53* gene. (**A**) Sanger sequencing of codons 727–735 of wild-type (WT) and c.730G > A (p.G244S) mutant *TP 53* genes. (**B**) Schematic diagram of *TP53* gene and p53 protein. The G244S mutation identified in this study was indicated with an arrow. TAD: transcription-activation domain; PRD: proline-rich domain; DBD: DNA-binding domain; NLS: nuclear localization signal; OD: homo-oligomerization domain. (**F**) Conservative analysis of the Glycine 244-containing portion in p53 protein. The Glycine 244 was highlighted.

To understand the genetic basis of this family, we performed exome sequencing on the proband and her parents^28^. As a result, we generated an average of 55.6 million reads that passed the quality assessment and were aligned to the human reference sequence. The mean sequencing depth was 64, and an average of 97.5% sequences was covered by more than 10 times. We identified an average of 40,934 variants, and an average of 10,194 NS/SS/Indel (non-synonymous/splice acceptor and donor site/ insertions or deletions) variants was located in the coding regions. After filtering common variants in the databases, we identified 57 gene mutations shared by the proband and her mother, including a *TP53* mutation (c.730G > A; p.G244S) (Fig. 3A).

As *TP53* is mutated in more than 50% tumors and is the major causative gene for Li Fraumeni syndrome, we analyzed the co-segregation of this variant by polymerase chain reaction (PCR) and direct sequencing in this family. As shown in Fig. 2A,B, G244S *TP53* mutation was identified in all available patients. This mutation was also identified in an unaffected individual (III: 3), a 6-year-old boy. Therefore, G244S *TP53* mutation co-segregated in this family. Patients (or their parents, if younger than 14 y) were informed consent for the molecular testing.

G244S mutation occurred in the hot spot mutation areas of *TP53* gene (Fig. 3B). Glycine 244 was conserved among species from drosophila to human (Fig. 3C). G244S alteration was predicted to be damaging as assayed with functional prediction tools, including PolyPhen-2 (http://genetics.bwh.harvard.edu/pph2/), Mutation Taster (http://www.mutationtaster.org/), Pmut (http://mmb2.pcb.ub.es:8080/PMut/) and SIFT (http://sift.jcvi.org).

In the International Agency for Research on Cancer (IARC) database (http://www.iarc.fr), there is a LFS family from Malaysia carried the G244S *TP53* mutation. Nevertheless, there seems no relationship between Malaysia family and the one reported here. Rather, the G244S seems to be a *De Novo* mutation occurred in patient I: 1, as his parents and siblings survived more than 65 years old with tumor-free.

### G244S mutation disrupted p53 function

**Figure 4:**
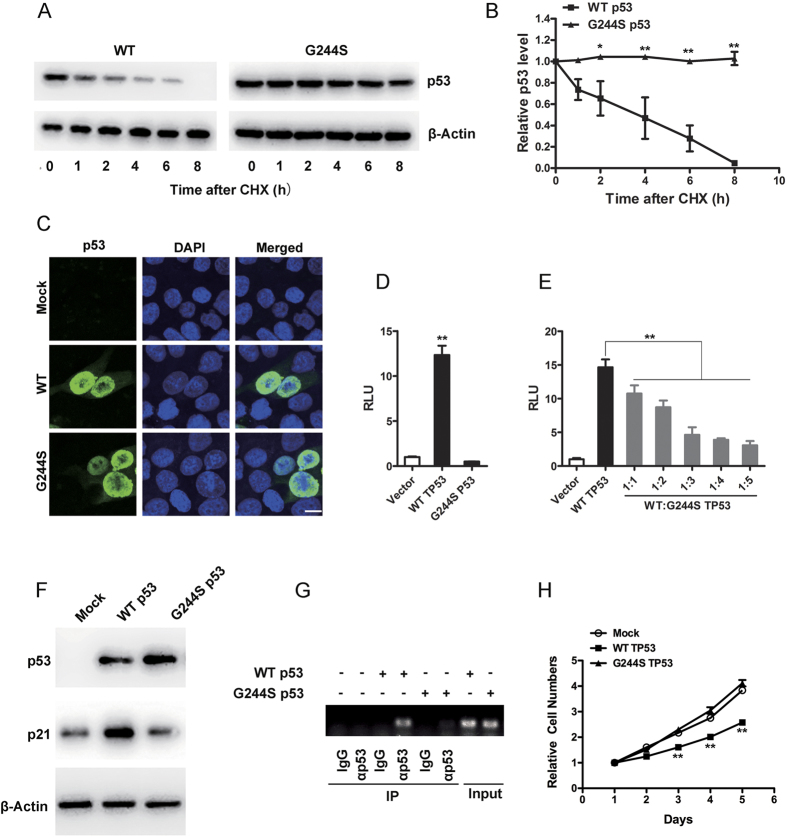
G244S mutation disrupted the p53 function. (**A**) G244S mutation increased the half-life of p53. *TP53*-deficient HCT116 cells were transiently transfected with plasmids expressing either the WT or G244S p53. 48 h later, cells were treated with cycloheximide (CHX) for 0, 1, 2, 4, 6 and 8 h. Equal amounts of whole cell lysates were analyzed by Western blot with a p53 antibody. Actin was used as an internal control. (**B**) The graph illustrates the quantification of WT and G244S p53 by densitometry of triplicate experiments (mean ± SEM). *p < 0.05, **p < 0.01 by post hoc Bonferroni *t* test. (**C**) The subcellular localization of WT and G244S p53 proteins as analyzed with immunofluorescence. Scale bar = 10 μm. (**D**) Transcriptional activities of WT and G244S p53 as determined by luciferase activity assays. The luciferase reporter plasmid *p21*-Luc were transiently transfected into *TP53*-deficient HCT116 cells in combination with WT or G244S p53 expressing plasmids. **p < 0.01 by post hoc Dunnett’s *t* test (**E**) The dominant negative effect of G244S p53 on WT p53. Increasing amount of G244S p53-expressing plasmid was cotransfected with a fixed amount of WT p53-expressing plasmid and the *p21*-Luc into *TP53*-deficient HCT116 cells. The basal level of luciferase was set as 1. Data from all other transfection are presented as fold induction above this level. Each value was the mean ± SEM of three replicates from a single assay. The results shown were representative of three independent experiments. **p < 0.01 by post hoc Bonferroni *t* test. (**F**) The induction of p21 after transfection of WT or G244S p53-expressing plasmids in *TP53*-deficient HCT116 cells. The protein levels of p53, p21 and β-actin was analyzed with immunoblotting. (**G**) The ability of WT and G244S p53 to bind corresponding DNA as assayed with a chromatin immunoprecipitation (ChIP) assay in *TP53*-deficient HCT116 cells. (**H**) The growth curve of SaoS2 cells stably transfected with WT or G244S *TP53*, as measured with a MTT assay. The cell numbers at day 1 was set to 1. **p < 0.01, post hoc Bonferroni *t* test.

G244S mutation significantly increased the half-life of p53 protein. As shown in Fig. 4A,B, wild-type (WT) p53 degraded rapidly, with a half-life of ~3 h. However, no significant degradation of G244S p53 was detected in 8 h after cycloheximide treatment. Both WT and G244S p53 proteins were localized in the nuclei (Fig. 4C). To study the functional consequences of G244S mutation, WT or G244S p53-expressing constructs were co-transfected with a luciferase construct under the control of *p21* promoter into *TP53*-deficient HCT116 cells. As shown in Fig. 4D, WT, but not G244S p53 elicited a significant increase in luciferase activity, which was also observed in other cell lines such as SaoS-2 and H1299 cells (data not shown).

To see if G244S p53 has dominant negative effect on the WT p53, the *p21*-luciferase construct was co-transfected with WT p53-expressing plasmid and increased doses of G244S p53-expressing constructs. Although high doses of G244S p53 inhibited the transactivity of WT p53, only minimal inhibition was observed when they were at a 1:1 ratio (Fig. 4E).

The *TP53*-deficient HCT116 cells were transfected with WT and G244S *TP53* gene, and the protein levels of p53 and p21 were assayed with Western blot. As shown in Fig. 4F. WT p53 significantly increased the p21 level, whereas G244S p53 showed no effect. As Glycine 244 was located in the DNA-binding domain of p53, we performed a chromatin immunoprecipitation (ChIP) assay in the *TP53*-deficient HCT116 cells to see if G244S mutation disrupted the DNA-binding activity of p53. As shown in Fig. 4G, the ability of p53 to pulldown *p21* promoter was disrupted by G244S mutation.

The p53 protein potently inhibited cell growth. To study the physiological consequence of G244S mutation, WT and G244S *TP53* were stably transfected into *TP53*-deficient SaoS-2 cells, and their growth curve was assayed. As shown in Fig. 4H, transfection of WT *TP53* significantly suppressed the cell growth, which is absent when G244S *TP53* was transfected.

### Loss of wild-type *TP53* in the tumor samples

**Figure 5:**
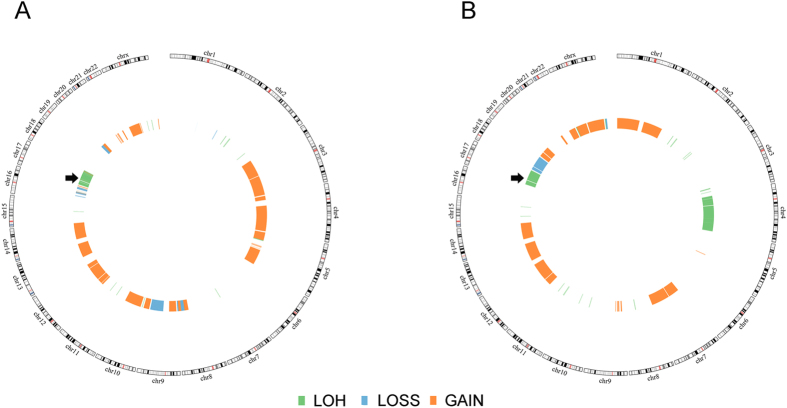
The Copy Number Variations (CNVs) identified in the tumor samples from III:4 (A) and III:5 (B). CNVs were analyzed with an Illumina Human Cyto SNP12, followed by Illumina’s Genome Studio Genotyping Module. The arrows indicated loss-of-heterozygosity (LOH) of chromosome 17, where *TP53* gene was located.

Consistent with most Li Fraumeni families, the tumor onset age became younger with successive generations. It has been suggested that the anticipation pattern may result from increased DNA copy-number variations (CNVs) with successive generations^29^. However, this phenomena was not verified in a whole genome sequencing study^30^. We performed karyotype as well as CNVs analysis on this family, and did not identify tumor-associated chromosome abnormality or CNVs. We did not either see a trend of increase in the CNVs numbers with successive generations (data not shown). We also performed CNVs analysis on the tumor samples from III: 4 and III: 5, there were 95 and 63 CNVs in the tumor samples from III: 4 and III: 5, respectively, covering about 50% of the chromosome (Fig. 5).

About 50% tumors from the LFS patients showed loss-of-heterozygosity (LOH), in which the functional wild-type *TP53* was lost. When checking the CNVs from III: 4 and III: 5’s tumor samples, we found that both tumors have two identical copies of chromosome 17 where *TP53* gene was located (Fig. 5). We therefore performed a PCR-Sanger sequencing and found the G244S *TP53* mutation was homozygous in the tumor sample, in contrast to the heterozygous status in the germline. Quantitative PCR analysis indicated that there were two copies of mutant *TP53* gene in both tumor samples.

In summary, we have identified, to our best knowledge, the first Li Fraumeni syndrome family in China. Six family members were affected with various tumors. A *TP53* mutation (G244S) co-segregated with the tumor phenotype within this family. Functional analysis indicated that G244G mutation disrupted the transactivity, DNA-binding and cell growth inhibition activity of p53 proteins. The identification of germline *TP53* mutation has important clinical implications for this family. Individuals with a deleterious *TP53* germline mutation have an approximately 90% lifetime risk of developing cancer, and 50% occurring before the age of 40 years. We have informed the patients (or their parents, if younger than 14 y) with consent for the *TP53* mutation. The mutation carriers are suggested to performed regular check according to the National Comprehensive Cancer Network (NCCN) guidelines. The carriers were also strongly recommended to perform pre-implantation genetic diagnosis if they get pregnancy in the future.

Considering the large population in China and the high risk of *TP53* germline carrier to develop cancer, the prevalence of germline *TP53* mutation in various tumor patients should be surveyed, and the possibility to perform *TP53* mutation test in high-risk individuals should be considered in China. Indeed, Cao *et al*. recently identified eight *TP53* mutations in non-LFS/non-LFL breast cancer families from China. Five of them are located in introns, and three of them are in the protein encoding region (L188P, R72P and S215_Y220del)^31^.

## Methods

### Patients

The proband was identified in the Department of Neurosurgery at Xiangya Hospital, Central South University of China. Nine subjects in her family were included in this study. Genomic DNA was extracted from peripheral blood leukocytes, and genomic DNA of tumors from the proband and her younger brother was also extracted. The study was approved by the Ethics Committee of Xiangya Hopsptal, and signed informed consent was obtained from each of the subjects (or their parents if younger than 14 y). All methods were performed in accordance with approved guidelines.

### Exome sequencing

For each sample, 3 μg of genomic DNA was sonicated into fragments, and used to construct a paired-end sequencing library with the Agilent SureSelect Target Enrichment System. Exome capture was performed with the Agilent SureSelect Human All Exon kit. Each sample was sequenced on an Illumina HiSeq2000 instrument. The sequenced reads were aligned to the human genome reference (UCSC hg 19 version) using Burrows-Wheeler Aligner (BWA). Reads qualities were recalibrated using Genome Analysis Toolkit (GATK). Picard 1.14 was used to flag duplicate reads. GATK IndelRealigner was used to realign reads around insertion/deletion sites. The Single Nucleotide Variants (SNVs) and small insertions and deletions (InDels) were generated with GATK Unified Genotyper and in parallel with the SAMtools pipeline. The called SNVs and Indels were annotated with ANNOVAR^32^,^33^,^34^,^35^,^36^,^37^.

### Plasmids and antibodies

The coding region of human *TP53* was amplified using polymerase chain reaction (PCR) and cloned into pcDNA3.1. The *p21*-luciferase reporter plasmid (*p21*-Luc) was a kind gift from Professor Peter Kaiser (University of California at Irvine)^38^. The G244S mutation was engineered using the QuikChange mutagenesis kit (Stratagene, La Jolla, CA) according to the manufacturer’s instructions. All the constructs were confirmed by Sanger sequencing. Antibodies against p53 and p21 were purchased from Cell Signaling Technology.

### Immunofluorescence staining

*TP53*-deficient HCT116 cells (a gift from Dr. Bert Vogelstein) were plated on coverslips and transfected with wild-type (WT) or G244S p53-expressing plasmids. At 36 h after transfection, cells were fixed in 4% paraformaldehyde for 30 min, followed by treatment with 1% Triton X-100 for 1 h at room temperature. Cells were then incubated with blocking solution (PBS, 3% bovine serum albumin, 5% goat serum) for 1 h. Incubation with p53 mouse antibody (1:400 dilution) was performed at 4 °C overnight. After being washed with PBS, fluorescence-labeled secondary anti-mouse antibodies (1:400 dilution; Invitrogen, Carlsbad, CA) were incubated for 2 h at room temperature in a dark room. After incubation with 4,6-diamino-2-phenylindole (DAPI; Invitrogen, Carlsbad, CA) for 5 min, cells were mounted in Fluoromount medium (Sigma, St Louis, WA), and fluorescence images were examined with a laser scanning confocal system installed on a Carl Zeiss microscope (Zeiss, Gottingen, Germany) with a 63× oil immersion objective.

### Luciferase reporter assay

*TP53*-deficient HCT116 cells were transfected with 5 ng of *p21*-luciferase reporter plasmid and 20 ng of expression constructs for wild-type (WT) or G244S p53. At 48 h after transfection, cells were washed with PBS and lysed with Reporter Lysis Buffer (Promega, Madison, WI). The extracts were assayed for luciferase activity using the Luciferase Assay System (Promega, Madison, WI) according to the manufacturer’s protocol. All reporter assays were conducted at least three times and performed in triplicate on different days using different batches of cells.

### Western blot analysis

*TP53*-deficient HCT116 cells were transfected with 200 ng of WT or G244S p53-expressing plasmids in 12-well plates. At 48 h after transfection, cells were lysed in 2XSDS loading buffer, containing 1 mM phenylmethanesulfonyl fluoride (PMSF) (Sigma, St Louis, WA), and 0.2 mM β-mercaptoethanol supplemented with protease inhibitor cocktail (Sigma, St Louis, WA). The lysate containing the same total protein was separated on 12% sodium dodecyl sulfate-polyacrylamide gel electrophoresis, transferred onto a polyvinylidene fluoride membrane and subjected to immunoblotting with p53 mouse antibody and p21 rabbit antibody. β-actin acts as a loading control.

To analyze the stability of WT and G244S p53, cycloheximide (Sigma, St Louis, WA, USA) was added to a final concentration of 100 μg/ml at 48 h after transfection. The cells were lysed at 0, 1, 2, 4, 6, 8 h after cycloheximide treatment, and the proteins were detected with Western blot.

### Real-time quantitative PCR

50 ng genomic DNA was used for real-time quantitative PCR using 1XSYBR Green PCR master mix (Takara Shuzo Co., Kyoto, Japan). The relative TP53 gene copy number in comparison with the cordon-bleu WH2 repeat protein gene (COBL) was calculated using the comparative cycles of threshold method.

### Cell proliferation assay

Human osteosarcoma SaoS-2 cells stably expressing WT or G244S p53 were plated onto 96-well plates, and the plates were harvested at day 1, 2, 3, 4, and 5. 20 μl of 5 mg/ml MTT (3-(4,5-dimethylthiazol-2-yl)-2,5-diphenyltetrazolium bromide) were added to each well, and carefully removed without disturbing the cells after incubation for 4 h at 37 °C. 150 μl dimethyl sulfoxide (DMSO) was added into each well and the plate was shaken at room temperature for 15 min. Absorbance was read at 590 nm with a reference filter of 620 nm.

### Chromatin immunoprecipitation assay

Chromatin immunoprecipitation (ChIP) assays were performed using Chromatin Immunoprecipitation Assay Kit (Millipore, Bedford, MA, USA) according to the manufacturer’s instructions. Chromatin was sonicated into 200–1000 bp fragments and precipitated with 2 μg of p53 mouse antibody or normal mouse IgG. Purified DNA was subjected to PCR amplification using primers spanning the p53-binding site on *p21* promoter (forward primer, 5′-AGCAGGCTGTGGCTCTGATT-3′; reverse primer, 5′-CAAAATAGCCACCAGCCTCTTCT-3′).

### Statistical analyses

A repeated measures ANOVA followed by unpaired *t* test was used to analyze the data for differences. All statistical analyses were performed using Prism 6 (GraphPad Software, San Diego, CA).

## Additional Information

**How to cite this article**: Hu, H. *et al*. Genetic and functional analysis of a Li Fraumeni syndrome family in China. *Sci. Rep.* **6**, 20221; doi: 10.1038/srep20221 (2016).
